# MCNN_MC: Computational Prediction of Mitochondrial Carriers and Investigation of Bongkrekic Acid Toxicity Using Protein Language Models and Convolutional Neural Networks

**DOI:** 10.1021/acs.jcim.4c00961

**Published:** 2024-08-12

**Authors:** Muhammad Shahid Malik, Yan-Yun Chang, Yu-Chen Liu, Van The Le, Yu-Yen Ou

**Affiliations:** † Department of Computer Science and Engineering, Yuan Ze University, Chung-Li 32003, Taiwan; ‡ Graduate Program in Biomedical Informatics, Yuan Ze University, Chung-Li 32003, Taiwan; § Department of Computer Sciences, Karakoram International University, Gilgit-Baltistan 15100, Pakistan

## Abstract

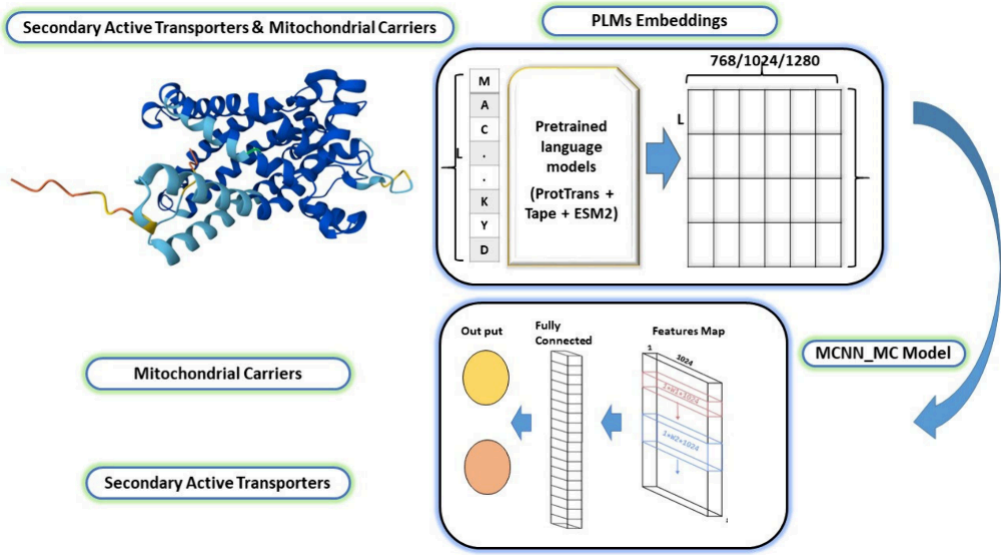

Mitochondrial carriers (MCs) are essential proteins that transport metabolites across mitochondrial membranes and play a critical role in cellular metabolism. ADP/ATP (adenosine diphosphate/adenosine triphosphate) is one of the most important carriers as it contributes to cellular energy production and is susceptible to the powerful toxin bongkrekic acid. This toxin has claimed several lives; for example, a recent foodborne outbreak in Taipei, Taiwan, has caused four deaths and sickened 30 people. The issue of bongkrekic acid poisoning has been a long-standing problem in Indonesia, with reports as early as 1895 detailing numerous deaths from contaminated coconut fermented cakes. In bioinformatics, significant advances have been made in understanding biological processes through computational methods; however, no established computational method has been developed for identifying mitochondrial carriers. We propose a computational bioinformatics approach for predicting MCs from a broader class of secondary active transporters with a focus on the ADP/ATP carrier and its interaction with bongkrekic acid. The proposed model combines protein language models (PLMs) with multiwindow scanning convolutional neural networks (mCNNs). While PLM embeddings capture contextual information within proteins, mCNN scans multiple windows to identify potential binding sites and extract local features. Our results show 96.66% sensitivity, 95.76% specificity, 96.12% accuracy, 91.83% Matthews correlation coefficient (MCC), 94.63% F1-Score, and 98.55% area under the curve (AUC). The results demonstrate the effectiveness of the proposed approach in predicting MCs and elucidating their functions, particularly in the context of bongkrekic acid toxicity. This study presents a valuable approach for identifying novel mitochondrial complexes, characterizing their functional roles, and understanding mitochondrial toxicology mechanisms. Our findings, that utilize computational methods to improve our understanding of cellular processes and drug-target interactions, contribute to the development of therapeutic strategies for mitochondrial disorders, reducing the devastating effects of bongkrekic acid poisoning.

## 1 Introduction

**Figure 1:**
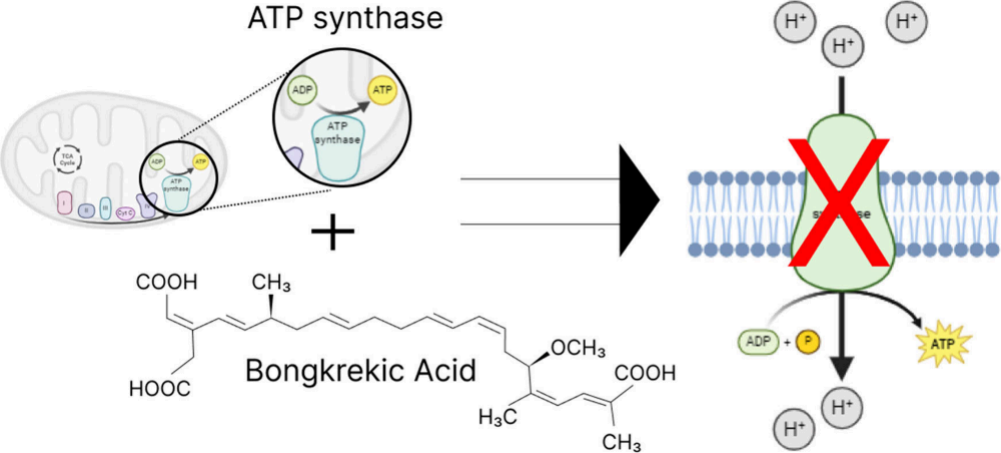
Illustrates the inhibition of ADP/ATP exchange by bongkrekic acid and its consequent impact on ATP production. This figure visually demonstrates how bongkrekic acid binds to the ADP/ATP carrier, preventing the normal exchange process and disrupting cellular energy production.

**Figure 2:**
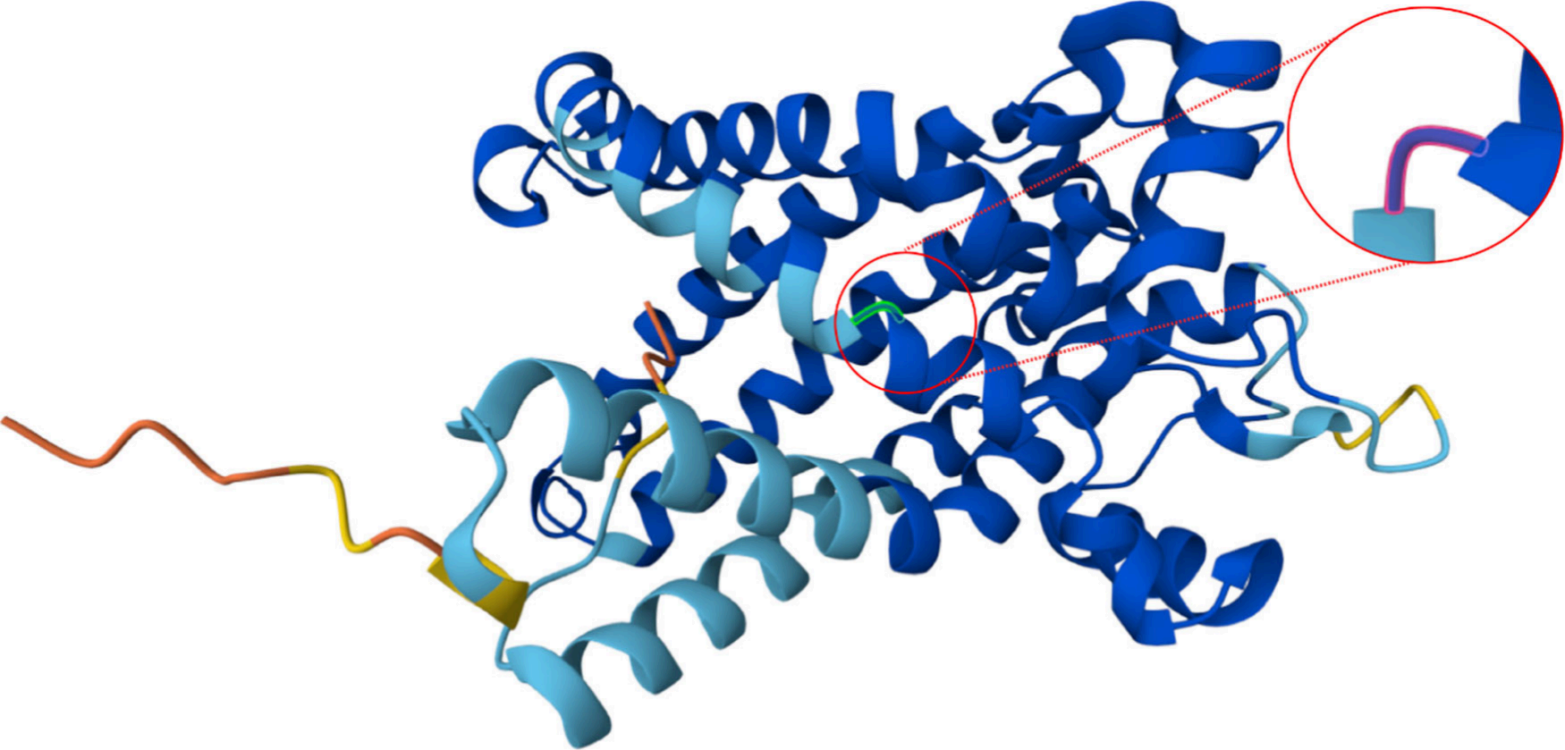
Reveals the three-dimensional structure of the G2QNH0 protein, highlighting the ADP/bongkrekic acid binding site. This structural representation shows how bongkrekic acid interferes with ADP/ATP translocase activity, providing insights into the molecular mechanisms underlying its toxic effects.

Mitochondrial carriers (MCs) are integral membrane proteins that transport metabolites across mitochondrial membranes and play a crucial role in metabolism and energy production.^1,2^ Among these carriers, the ADP/ATP carrier (AAC) plays a critical role in the exchange of cytosolic ADP for mitochondrial synthesized ATP, thereby maintaining cellular energy homeostasis.^3,4^ The AAC is also a target for the potent toxin bongkrekic acid, produced naturally by the bacterium burkholderia gladioli pathovar cocovenenans.^5−7^ According to Figure 1, bongkrekic acid inhibits the ADP/ATP exchange process which affects the production of ATP. In addition to disrupting cellular energy production and preventing normal exchanges, bongkrekic acid binds to the ADP/ATP carrier. By binding to the AAC in a cytosolic conformation, bongkrekic acid prevents the exchange of ADP and ATP across the mitochondrial membrane, resulting in energy depletion and cell death.^8,9^ As illustrated in Figure 2, the G2QNH0 protein has a three-dimensional structure that highlights a binding site for ADP/bongkrekic acid. Bongkrekic acid interferes with the ADP/ATP translocase activity through this structural representation, providing insights into its toxic effects.

Bongkrekic acid has been implicated in several foodborne outbreaks, including a recent incident in Taipei, Taiwan, where the bacterium Burkholderia gladioli produced a powerful toxin that caused severe poisoning cases. Similarly, bongkrekic acid poisoning has been a long-standing issue, with reports of countless deaths in Indonesia caused by traditional coconut-fermented cakes contaminated with the toxin dating back to 1895.^10^ These incidents underscore the importance of understanding the interactions between toxic substances and their biological targets. They also highlight the ability to accurately predict the presence of such substances in food preparation environments.

Over the past few years, there has been an increasing interest in targeting mitochondrial carriers, such as the AAC, as therapeutic interventions in disorders associated with mitochondrial dysfunction.^11,12^ In several studies, the potential of pharmacologically modulating mitochondrial carriers has been highlighted, since mitochondria regulate ATP production and consumption within cells.^3,13^ The mitochondrial adenine nucleotide translocase (ANT), which facilitates DNA replication and synthesis, has also proven to be a potential drug target for maintaining mitochondrial function and cell viability.^1,4^

It is imperative to develop accurate computational methods for predicting and characterization of mitochondrial complexes in light of their significance in cellular function and the relevance of mitochondrial diseases and toxin-mediated diseases. Although many mitochondrial carriers are incompletely annotated, bioinformatics faces several challenges. These include limited functional data, incomplete structural information, and inconsistent nomenclature and classification.^14,15^ A bioinformatics approach that leverages the increasing availability of protein sequence and structural data can provide powerful tools for addressing this challenge.^16,17^ In this study, we intend to address these challenges by applying advanced computational methods to predict MCs within the broader class of secondary active transporters using a combination of pretrained Protein Language Models (PLMs) and multiwindow scanning Convolutional Neural Networks (mCNNs). This study focuses on the ADP/ATP carrier and its interaction with bongkrekic acid to gain insight into the structural determinants of binding specificity and the molecular mechanisms responsible for toxicity. Using ProtTrans embeddings,^18^ we enhanced the classification of mitochondrial carriers and identified potential bongkrekic acid drug targets. As a result of integrating ProtTrans embeddings into our computational framework, we improve our understanding of mitochondrial biology and accelerate drug discovery efforts for mitochondrial dysfunction-related diseases by leveraging the wealth of information encoded within protein sequences.

Based on recent advances, Bao and colleagues^19^ introduced a complex-valued polynomial model for identifying protein acetylation sites as an innovative computational approach for analyzing protein functions across different biological systems. Using this model, we could better understand protein modifications and their consequences. Similarly, they developed the ’Oral_voting_transfer’ model,^20^ a method for classifying oral microorganism functional proteins that offer novel insights into microbial ecology and pathogenesis. In another related work, using a deep forest algorithm ’Golgi_DF’^21^ to classify Golgi proteins offers a robust framework for predicting Golgi apparatus protein functions, potentially influencing cellular trafficking and disease research. Overall, these studies provide a better understanding of complex biological systems and computational tools for analyzing biological data Our approach using recent methodologies provides insights into Bongkrekic Acid toxicological mechanisms as well as enhanced mitochondrial carrier prediction accuracy.

We employed the multiple window scanning model, this model consistently predicted membrane protein functions and secondary active transporters, demonstrating better performance in bioinformatics analysis based on previous studies.^22−24^ Further, evolutionary and contextual information can be captured within protein sequences using PLMs, and using different window sizes and convolution neural networks, the mCNN extracts local structural patterns around potential binding sites. The combination of these techniques that we realized in this work explore how bongkrekic acid interacts with ADP/ATP carriers and distinguishes MCs from other secondary active transporters. In addition to identifying novel mitochondrial complexes and clarifying their functional roles, this study provides the framework for understanding mitochondrial toxins as well as developing new therapeutic strategies for mitochondrial disease, which will advance the development of new drugs for mitochondrial diseases.

## 2 Materials and Methods

**Figure 3:**
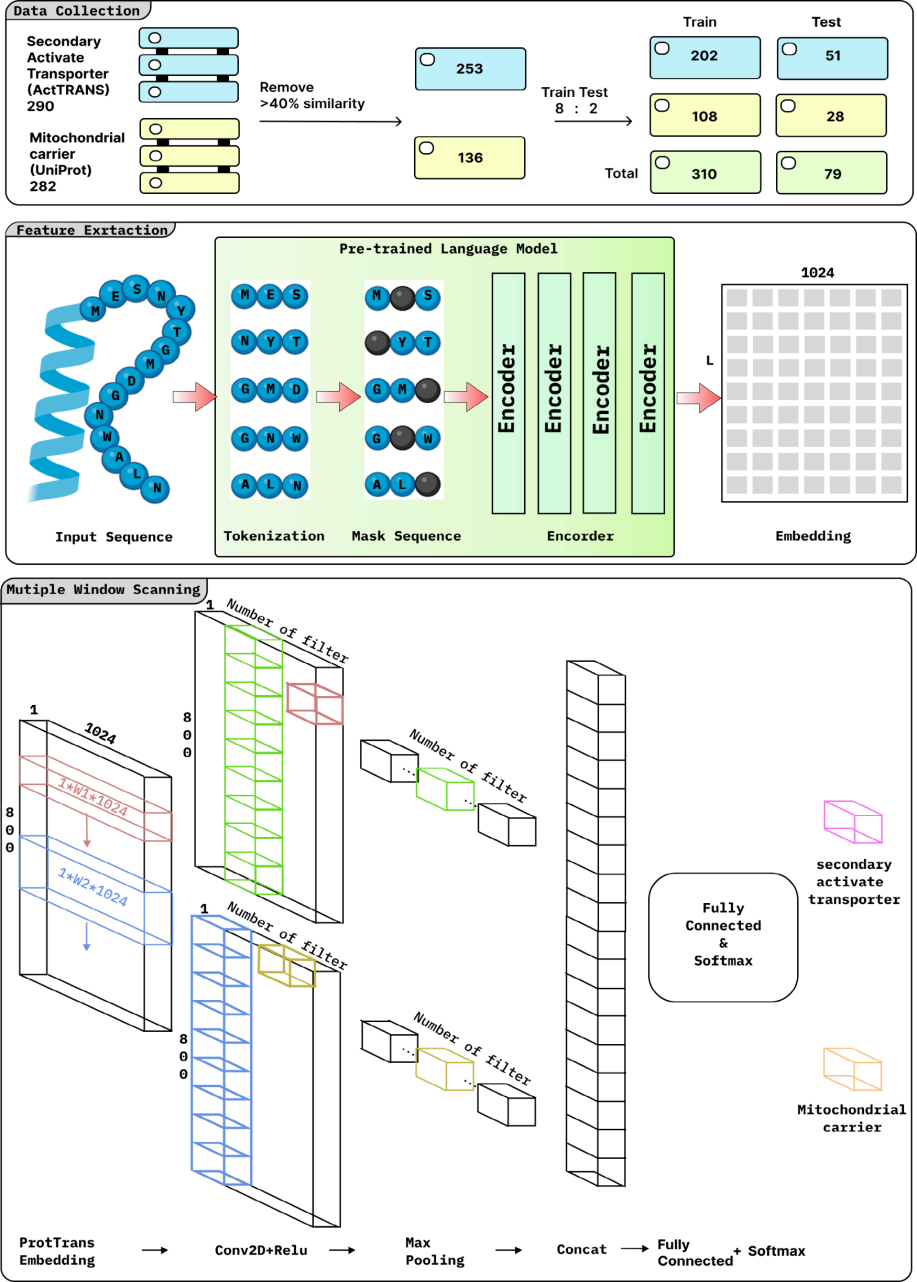
Workflow of the MCNN_MC model.

Multiple window deep learning algorithms and bioinformatics techniques are employed to classify and analyze mitochondrial carriers. Figure 3: The workflow of the MCNN_MC model, which starts with input data as MCs and SATs. These data are preprocessed to reduce redundancy by clustering similar sequences. Next, three distinct pretrained Protein Language Models (PLMs) are used to generate embeddings. The embeddings, which encapsulate the intricate features and contextual information on the sequences, are then fed into our deep learning model, where a multiple-window convolutional neural network (mCNN) categorizes mitochondrial carriers (MCs) from secondary active transporters.

### 2.1 Data Collection

To explore mitochondrial carriers (MCs) as potential targets for bongkrekic acid, data sets are curated from UniProt database.^25^ In the data set, GO:0006839 for mitochondrial transport is used to describe the broad class of mitochondrial transporters. Among this main class of ADP/ATP carriers are proteins essential to cell metabolism and signaling. Since secondary active transporters (SATs) play a major role in cellular metabolism and are potential drug targets, we included the GO term GO:0015291 to indicate that secondary active transporters are involved in the data set.

The data set included 282 sequences for the mitochondrial carriers class, while the secondary active transporters class contained 290 sequences. By eliminating redundancy and ensuring that the data set contains a diverse but representative set of sequences, the CD-HIT step is crucial for subsequent analysis.^26^ In the data sets, after applying CD-HIT with a threshold of 40% similarity, the number of distinct sequences of mitochondrial carrier class and SATs class are reduced to 136 and 253, respectively. In both data sets, 80% of the sequences were allocated to the training set and the rest to the validation set, thereby ensuring a representative distribution of classes. In the training set, 108 sequences were assigned to the mitochondrial carriers class, while 202 sequences were assigned to the secondary active transporters class. In the validation set, 28 mitochondrial carriers and 51 secondary active transporters were analyzed, as shown in Table 1.

**Table 1:** Statistical Analysis of Mitochondrial Carriers (MCs) and Secondary Active Transporters (SATs)

Class Name	Original	Similarity <40%	Train	Test
Secondary Active Transporter	290	253	202	51
Mitochondrial Carrier	282	136	108	28
**Total**	**572**	**389**	**310**	**79**

### 2.2 Feature Extraction

We used embeddings from pretrained language models (PLMs) to capture the complex semantic and contextual information inherent in protein sequences, which improves downstream bioinformatics and protein analysis. Our model studies embeddings generated by three state-of-the-art PLMs: ProtTrans, ESM2, and TAPE.

The field of bioinformatics has been transformed by pretrained language models that provide nuanced representations of protein sequences. Through the use of extensive protein databases, these models can encode rich semantic and contextual information that is difficult to convey using traditional methods such as one-hot encoding and multiple sequence alignment. These embeddings provide us with a better understanding of protein structure and function that, allows us to make more accurate predictions and analyses.

Recent advances in protein pretrained language model embeddings have revolutionized protein analysis by capturing intricate patterns and semantic information within protein sequences. ProtTrans stands out among these^18^ models for its five transformer-based models, including BERT, Albert, and XLNet. As a result of this ensemble approach, ProtTrans generates rich, context-aware representations of protein sequences, which is useful for functional annotations, structural predictions, and other bioinformatics analyses. The ProtBert model is an adaptation of the BERT architecture specifically designed for protein sequences.^16^ This allows it to perform protein-related tasks such as classification, function prediction, and structure prediction. Furthermore, models like TAPE (The Annotated Protein Embedding) have been instrumental in pretraining embeddings that capture diverse protein properties and functions.^27,28^ The use of recurrent neural networks (RNNs) in protein sequence analysis has also been demonstrated through models such as UniRep (Universal Representation).^29^ By leveraging these pretrained language model embeddings, bioinformatics and protein-related research can extract and analyze protein features more accurately.

By integrating advances in pretrained language model embeddings, we will be able to enhance our classification strategy, enhancing our ability to classify and analyze protein sequences more accurately. The ProtTrans model, as introduced by Elnaggar et al.,^18^ has emerged as a powerful tool for understanding life’s language through self-supervised learning. ESM2, introduced by Rives et al.,^30^ has shown remarkable capabilities in capturing intricate sequence patterns and extracting meaningful features. Additionally, the TAPE model proposed by Rao et al.^28^ has succeeded in numerous protein-related tasks. By incorporating these state-of-the-art techniques, we aim to compare their effectiveness and identify the most appropriate method for our classification task.

#### 2.2.1 ProtTrans Embedding Model for Protein Languages

The ProtTrans approach^18^ stands out as a cutting-edge method for protein sequence analysis as it captures intricate features and semantic information embedded within amino acid sequences. This study uses the ProtTrans model “Rostlab/prot_t5_xl_half_uniref50-enc,″ which contains 24 layers and approximately 1.2 billion parameters. By employing a 1024-dimensional embedding scheme to represent amino acids, ProtTrans facilitates rich and expressive representations of protein sequences by leveraging pretrained language models. This high-dimensional embedding strategy enables ProtTrans to better understand protein structures, functions, and interactions. Utilizing self-supervised learning techniques, ProtTrans extends beyond traditional sequence analysis methods, allowing it to extract hierarchical representations of complex amino acid interactions. Furthermore, ProtTrans has been trained on extensive data sets that include millions of protein sequences, ensuring robustness and adaptability to diverse biological contexts. Through sophisticated self-supervised learning objectives, including amino acid prediction and sequence reconstruction, ProtTrans can effectively learn from unlabeled data during its training process. This approach offers unprecedented insights into proteins’ functional properties and evolutionary dynamics, with implications for a wide range of biological and biomedical studies.

#### 2.2.2 ESM2 Embeddings

ESM2 model^30^ embeddings represent biological sequences generated by the ESM2 model, a sophisticated analytical framework for protein sequences. We use the ESM2 model composed of 33 layers, over 650 million parameters, and 1280-dimensional embeddings. To generate embeddings, sequences are tokenized with the ESM2 tokenizer and then passed through the model. It is possible to customize embeddings based on specific use cases by averaging or pooling tokens or layers. As a result, ESM2 embeddings enable downstream tasks in bioinformatics and protein analysis. These tasks include classifying, clustering, and calculating similarity. During our analysis, we use ESM-2 embeddings to provide context and encode complex patterns. Incorporating ESM-2 into our research will improve our ability to comprehend these critical biological functions and increase the depth and complexity of our molecular analysis.

#### 2.2.3 Tape Embeddings

The TAPE (Tasks Assessing Protein Embeddings), Transformer Architecture for Protein Embedding^28^ represents a pioneering approach to the analysis of protein sequences, renowned for its robustness and versatility. With TAPE, amino acids are encoded in rich and expressive representations using a 768-dimensional embedding scheme that captures the essence of protein sequences. The architecture of TAPE is based on transformer-based models, which are renowned for their effectiveness in natural language processing. It encapsulates essential sequence information while facilitating efficient processing and analysis. By capturing long-range dependencies within protein sequences, TAPE extracts nuanced features to understand protein structure and function. In addition, TAPE has extensive pretraining on large protein sequence data sets, so its representations are robust and comprehensive. A key feature of TAPE is its ability to learn on protein sequences without labeling by using advanced self-supervised learning techniques, such as masked language modeling and next-sentence prediction. Through this approach, TAPE provides insight into how proteins function, evolve, and interact, which is relevant to drug discovery, bioengineering, and precision medicine.

### 2.3 Model Architecture

The mCNN-ETC model^22^ utilizes a multiple-window scanning technique to extract information about amino acid sequence structure and function. This deep learning approach leverages pretrained protein language model embeddings to gain a deeper understanding of evolutionary dynamics. mCNN employs multiple convolutional layers, each with a different window size, to provide comprehensive protein sequence analysis across a wide range of scales. During a convolution process, the 3D protein language embeddings are transformed into a 2D matrix of dimensions L x 1, where L indicates the sequence length and 1 indicates the number of filters. Through this approach, the model can capture diverse patterns and features present in the input sequences.

To analyze protein sequences across scales and resolutions, maximum pooling is applied to the L x 1 output after convolution. By pooling the convolutional output, the model can capture the most salient features effectively. Specific adjustments were made for protein sequence analysis tasks, including adjusting the number of classes to ensure that the model was suitable. To optimize the model’s performance during training, a cross-entropy loss function was selected. SoftMax activation functions were incorporated into the output layer to provide probabilistic interpretations of the model’s predictions. Combining these adjustments improves the capability of the model to identify protein sequences.

## 3 Performance Evaluation

To evaluate the effectiveness of our deep learning model, we needed a comprehensive set of metrics. These metrics show how well the model categorizes instances and provide insight into its overall performance. In this evaluation process, we used sensitivity, specificity, accuracy, Matthews correlation coefficients (MCCs), and F1-Score, which are commonly used to evaluate model performance in a variety of fields.^31−33^ The model’s sensitivity and specificity show its ability to accurately identify positive and negative instances, respectively, so that false positives and negatives can be minimized. Accuracy is a measure of how accurate the model is as a whole. Mathematical correlation coefficients (MCCs) provide nuanced insights into classification performance by accounting for false positives and negatives as well as true positives. Further, the F1-Score demonstrates that our model can identify true positives while minimizing false positives. Equations 1–5evaluate our model’s sensitivity, specificity, accuracy, MCC, and F1-Score.

Additionally, AUC-ROC was used to evaluate our deep learning model’s performance on the aforementioned evaluation metrics. As a single value calculated by plotting true positives versus false positives across different threshold levels, the AUC-ROC indicates a model’s discriminative power by separating positive from negative instances. The AUC-ROC, combined with sensitivity, specificity, accuracy, MCC, and F1-Score allows for a thorough evaluation of the model’s classification performance.
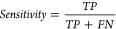
1
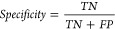
2
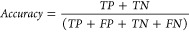
3
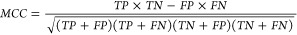
4
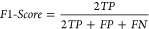
5TP represents correct positive identifications, TN indicates correct negative identifications, FP signifies incorrect positive identifications, and FN denotes incorrect negative identifications.

## 4 Results

The present study examined mitochondrial carriers as possible drug targets for bongkrekic acid, specifically ADP/ATP antiporters within the broader category of secondary active transporters. The analysis begins with the classification of mitochondrial carriers, which is followed by an examination of the ADP and ATP antiporters. Using 5-fold cross-validation, we evaluate the accuracy of our classification of mitochondrial carriers within secondary active transporters and antiporters responsible for the transport of ATP and ADP. In the Results and Discussion sections, we demonstrate the robustness and effectiveness of our computational model in predicting biologically significant protein functions.

Comparing mitochondrial carrier classification metrics across different window sizes reveals that window size has a significant impact on classification accuracy. For both 8 and 64 window sizes, sensitivity peaks at 0.9833, indicating their effectiveness in identifying true positives. In contrast, window size 2 achieves the highest specificity at 0.9498, indicating a strong ability to identify true negatives. However, as window sizes increase, specificity decreases, with a noticeable drop recorded at window sizes greater than 256, which recorded 0.8212. With a window size of 16, accuracy reaches 0.9612, achieving a balance between true positives and false negatives. The Matthews correlation coefficient (MCC), which takes into account both true and false positives and negatives, peaks at 16 with a value of 0.9196, indicating the best overall performance. Moreover, across all window sizes, the highest area under the curve (AUC) value of 0.9858 is recorded at a window size of 16. Based on these metrics, 16 is determined to be the optimal window size for mitochondrial carrier classification. Additionally, this window size provides the highest sensitivity, specificity, and AUC, indicating robust and reliable performance. Table 2 summarizes the results.

**Table 2:** Performance Comparison of Mitochondrial Carrier (MCs) Classification for Single Window Sizes

Window size	Sensitivity	Specificity	Accuracy	MCC	AUC
2	0.9619	0.9498	0.9516	0.8991	0.9815
4	0.9642	0.9468	0.9548	0.9050	0.9854
8	0.9833	0.9371	0.9548	0.9068	0.9832
**16**	**0.9738**	**0.9517**	**0.9612**	**0.9196**	**0.9858**
32	0.9738	0.9468	0.9580	0.9123	0.9812
64	0.9833	0.9324	0.9516	0.9003	0.9741
128	0.9666	0.9292	0.9419	0.8789	0.9719
256	0.9680	0.9213	0.9354	0.8660	0.9734

Table 3 shows that mitochondrial carrier classification metrics perform differently across different window combinations. When window sizes 4 and 16 are combined, this combination has excellent performance, demonstrating sensitivity 0.9738, specificity 0.9561, accuracy 0.9645, MCC 0.9266, and AUC 0.9830. Combining window sizes 4, 8, and 16 leads to an increase in sensitivity to 0.9833 and an increase in AUC to 0.9839, but accuracy is reduced to 0.9580 and MCC is decreased to 0.9141. Comparatively, combining window sizes 2, 4, 8, and 16 yields balanced results, with a sensitivity of 0.9666, specificity of 0.9525, accuracy of 0.9581, MCC of 0.9111, and AUC of 0.9816. Even though the combination of window sizes 4 and 16 performs well across all metrics, the combination of sizes 2, 4, 8, and 16 performs better. Using window sizes 2, 4, 8, 16, 32, and 64 results in robust performance, with 0.9667 sensitivity, 0.9183 MCC, and 0.9802 AUC. A larger window size, such as 128 or 256, results in higher specificity but lower sensitivity. In this example, using window sizes 2, 4, 8, 16, 32, 64, 128, and 256 results in an accuracy of 0.9580, a MCC of 0.9099, and an AUC of 0.9806. The optimal window combination for the study is 4 and 16, providing the highest accuracy, MCC, sensitivity, and specificity, ensuring robust and reliable model performance.

**Table 3:** Performance Comparison of Mitochondrial Carrier (MCs) Classification with Different Multiple Window Combinations

Multiple window combinations	Sensitivity	Specificity	Accuracy	MCC	AUC
**(4, 16)**	**0.9738**	**0.9561**	**0.9645**	**0.9266**	**0.9830**
(4, 8, 16)	0.9833	0.9420	0.9580	0.9141	0.9839
(2, 4, 8, 16)	0.9666	0.9525	0.9581	0.9111	0.9816
(2, 4, 8, 16, 32)	0.9738	0.9422	0.9548	0.9058	0.9821
(2, 4, 8, 16, 32, 64)	0.9667	0.9576	0.9612	0.9183	0.9802
(2, 4, 8, 16, 32, 64, 256)	0.9595	0.9537	0.9548	0.9036	0.9792
(2, 4, 8, 16, 32, 64, 128, 256)	0.9489	0.9642	0.9580	0.9099	0.9806

A comparison of mitochondrial carrier classifications for different filter sizes reveals a variety of impacts on the classification metrics. With a filter size of 64, the sensitivity is 0.9738, specificity is 0.9468, accuracy is 0.9580, MCC is 0.9123, and AUC is 0.9843, indicating robust performance. In the case of a filter size of 128, sensitivity decreases slightly, but specificity increases to 0.9576, resulting in an accuracy of 0.9612, MCC of 0.9183, and AUC of 0.9855 among the tested sizes, indicating a well-balanced and effective classifier. With a filter size of 256, the sensitivity is 0.9833, but the specificity is 0.9420, with an accuracy of 0.9580, MCC of 0.9141, and an AUC of 0.9839. As a result of providing 512 filters, we found that the sensitivity, specificity, accuracy, MCC, and AUC were 0.9643, 0.9469, 0.9548, 0.9050, and 0.9837, respectively, indicating high performance with a low MCC. A filter size of 1024 outperforms others when it comes to identifying true negatives, with the highest specificity and accuracy, along with sensitivity and MCC of 0.9619, and AUC of 0.9850. As shown in Table 4, the appropriate filter size for the study is 128, since it provides the highest level of sensitivity, specificity, accuracy, MCC, and AUC.

**Table 4:** Performance Comparison of Mitochondrial Carrier (MCs) Classifications with Different Filter Sizes

Filter size	Sensitivity	Specificity	Accuracy	MCC	AUC
64	0.9738	0.9468	0.9580	0.9123	0.9843
**128**	**0.9666**	**0.9576**	**0.9612**	**0.9183**	**0.9855**
256	0.9833	0.9420	0.9580	0.9141	0.9839
512	0.9643	0.9469	0.9548	0.9050	0.9837
1024	0.9619	0.9665	0.9613	0.9205	0.9850

**Figure 4:**
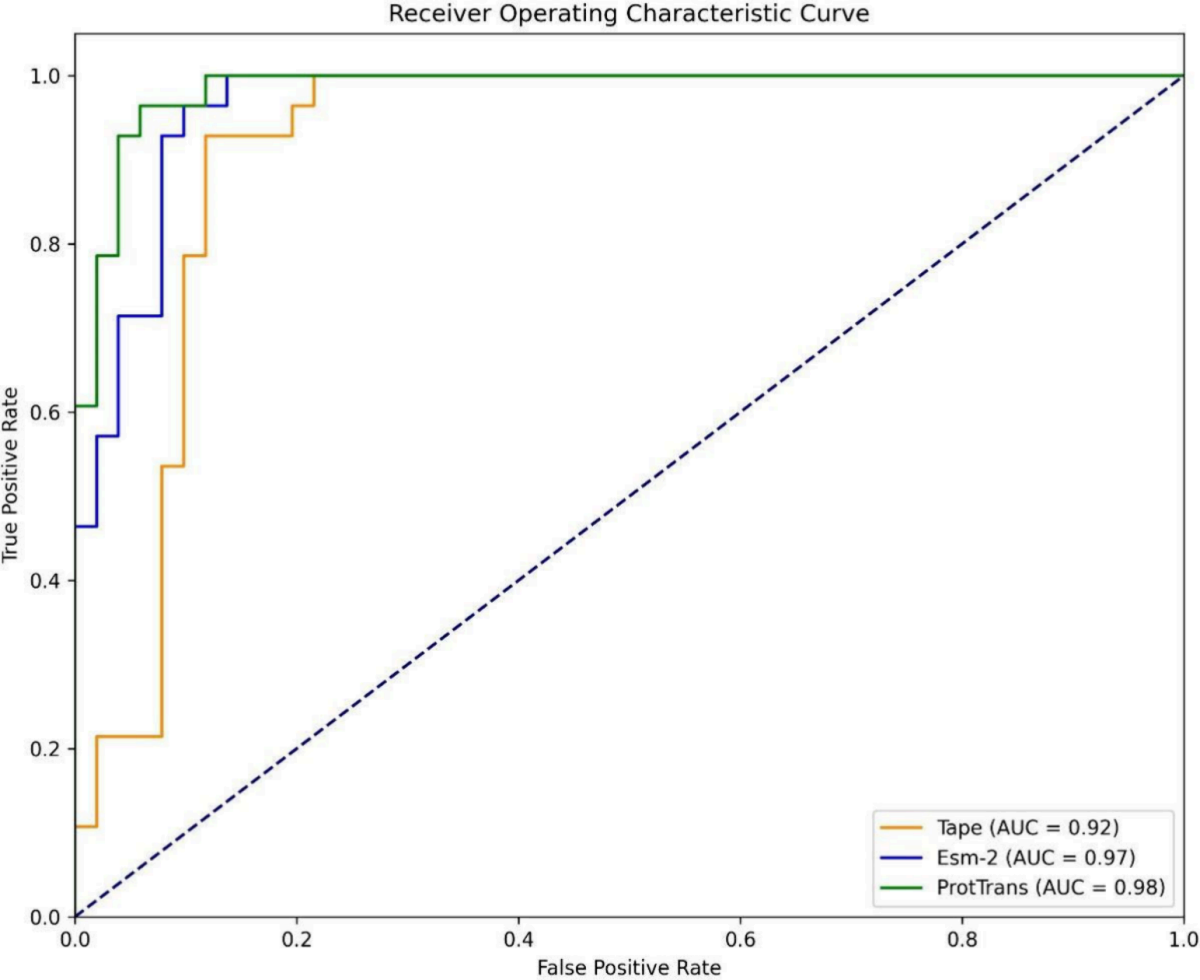
Comparison of the ROC curves with different protein language model feature sets for mitochondrial carrier’s classification.

Based on Table 5, an analysis of mitochondrial carrier classifications using different pretrained protein language model embeddings indicates that the classification metrics differ significantly. TAPE model has an accuracy of 0.9258, a MCC of 0.8517, and an AUC of 0.9482, but a low specificity of 0.8938. TAPE is effective in identifying true positives, but less effective in identifying true negatives. The ESM2 model has a sensitivity of 0.9928 and specificity of 0.9180, translating into an accuracy of 0.9451, an MCC of 0.8880, an F1 score of 0.9335 and an AUC of 0.9781. Overall, this model provides balanced performance with strong metrics. In comparison to other models, ProtTrans exhibits the highest performance with a sensitivity of 0.9666 and specificity of 0.9576, resulting in an accuracy of 0.9612, MCC of 0.9183, F1 score of 0.9463, and AUC of 0.9855. According to these metrics, ProtTrans provides the most accurate mitochondrial carrier classification, with the highest MCC and AUC values. Its robust and reliable performance identifies true positives as well as false negatives. Figure 4 illustrates the ROC curves for these comparisons.

**Table 5:** Performance Comparison of Mitochondrial Carrier (MCs) Classification Using 5-Fold Cross-Validation with Features from Different Pretrained Protein Language Models

Feature set	Sensitivity	Specificity	Accuracy	MCC	F1-Score	AUC
Tape	0.9833	0.8938	0.9258	0.8517	0.8950	0.9482
ESM2	0.9928	0.9180	0.9451	0.8880	0.9335	0.9781
**ProtTrans**	**0.9666**	**0.9576**	**0.9612**	**0.9183**	**0.9463**	**0.9855**

In addition to the 5-fold cross-validation, an independent test was conducted to evaluate the accuracy of mitochondrial carrier classifications using different pretrained language model features, as shown in Table 6. The Tape feature set showed sensitivity of 0.9286, specificity of 0.8824, accuracy of 0.8987, MCC of 0.7002, F1 score of 0.8667, and AUC of 0.9317. Despite its effectiveness in identifying true positives, the Tape feature set had a low specificity, and the overall performance metrics were inferior to those of the other feature sets. ESM2 has sensitivity of 0.9643, specificity of 0.9020, accuracy of 0.9241, MCC of 0.8441, F1 score of 0.9000, and AUC of 0.9671. The feature set demonstrated a more balanced and robust classifier. ProtTrans outperformed all other models, achieving the highest sensitivity, specificity, accuracy, MCC, F1 score, and AUC among them. Providing reliable and accurate results, ProtTrans excels at identifying both false positives and false negatives. As determined by this independent test, ProtTrans provides superior accuracy, MCC, and AUC as well as robust and reliable performance across all evaluation criteria.

**Table 6:** Performance of Mitochondrial Carrier (MCs) Classification on an Independent Test Set Using Features from Different Pretrained Protein Language Models

Feature set	Sensitivity	Specificity	Accuracy	MCC	F1-Score	AUC
Tape	0.9286	0.8824	0.8987	0.7902	0.8667	0.9167
ESM2	0.9643	0.9020	0.9241	0.8441	0.9000	0.9671
**ProtTrans**	**0.9643**	**0.9412**	**0.9494**	**0.8924**	**0.9310**	**0.9881**

**Figure 5:**
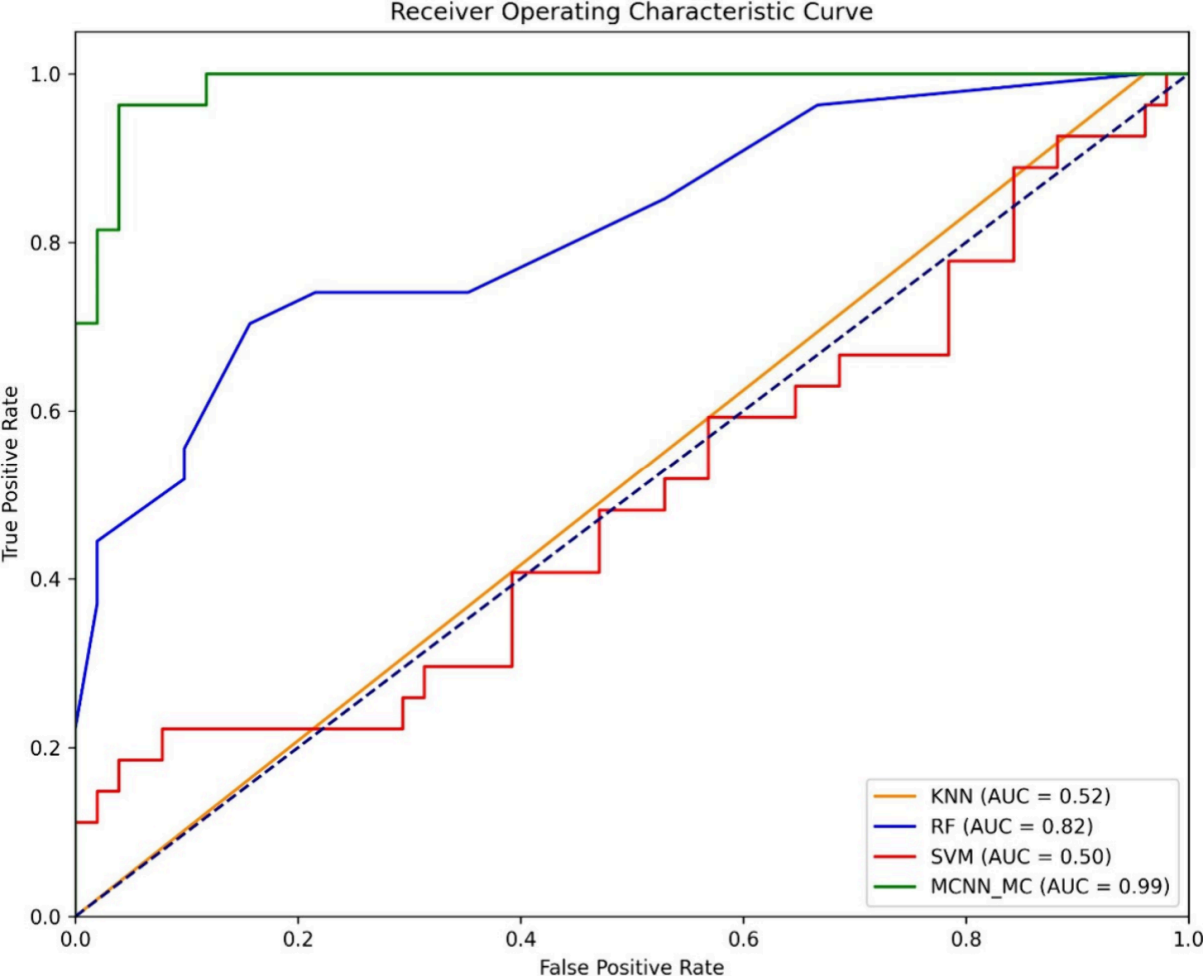
Comparisons of the ROC curves with MCNN_MC and different traditional machine learning models.

In Table 7, we compared MCNN_MC to other machine learning classifiers, including K-Nearest Neighbors (KNN), Random Forests (RF), and Support Vector Machines (SVM), for mitochondrial carrier classification, analyzing sensitivity, specificity, accuracy, MCC, F1-Score, and AUC. KNN has a sensitivity of 1.0 and a specificity of 0.0392 resulting in an overall accuracy of 0.3797, as well as an MCC of 0.1194, F1 score of 0.5263 and an AUC of 0.5196. While KNN identifies almost all positives, it struggles to identify negatives. While the RF model displayed better overall classification, its low sensitivity still caused it to face limitations. Such limitations include its sensitivity of 0.5714, specificity of 0.8823, accuracy of 0.7721, MCC of 0.4842, F1 score of 0.3529 and AUC of 0.8434. Using the SVM model, the AUC is 0.4397, sensitivity is 0.3214, accuracy is 0.6708, MCC is 0.2192, and specificity is 0.8627, indicating poor performance for both true positives and false negatives. According to our model MCNN_MC, achieved a sensitivity of 0.9643, a specificity of 0.9412, an accuracy of 0.9494, a MCC of 0.8924, a F1 score of 0.9310 and an AUC of 0.9881. In addition to identifying true positives and false negatives effectively, MCNN_MC demonstrated superior performance overall. As shown in Figure 5, the comparison of ROC curves illustrates the superior performance of MCNN_MC over other models.

**Table 7:** Comparison of Mitochondrial Carrier (MCs) Classifications with Different Machine Learning Models

Classification models	Sensitivity	Specificity	Accuracy	MCC	F1-Score	AUC
KNN	1.0	0.0392	0.3797	0.1194	0.5333	0.5196
RF	0.5714	0.8823	0.7721	0.4842	0.3529	0.8434
SVM	0.3214	0.8627	0.6708	0.2192	0.2222	0.4397
**MCNN_MC**	**0.9643**	**0.9412**	**0.9494**	**0.8924**	**0.9310**	**0.9881**

## 5 Discussion

MCNN_MC provides valuable insight into the classification of mitochondrial carriers (MCs) within the context of secondary active transporters. Utilizing protein language models and mCNN, we identified and analyzed the ADP/ATP carrier, which sheds light on its role in bongkrekic acid toxicity. Our approach has proven highly useful in classifying MCs, particularly the ADP/ATP carriers critical for cellular energy metabolism.

Our study investigated the optimal window size for our scanning multiple windows model, assessing which sequence lengths would be more advantageous. The best configuration was determined by exploring combinations of different windows based on these sizes. To improve model performance, several filter sizes were evaluated. According to cross-validation testing of protein language model embeddings, ProtTran embeddings outperformed TAPE and ESM-2 embeddings. In addition to cross-validation, an independent data set produced similar results. Compared with traditional machine learning models such as KNN, RF, and SVM, our model achieved a high sensitivity of 0.9666, an overall accuracy of 0.9612, and an AUC of 0.9855, indicating its robustness and effectiveness.

Our analysis highlights the potential toxicological implications of bongkrekic acid, highlighting its potential impact on mitochondrial function and cell homeostasis. Our findings underscore the importance of understanding the functional attributes of MC, particularly with respect to disease mechanisms and drug targeting. Beyond contributing to advancements in bioinformatics and protein analysis, our insights will aid future researchers in comprehending the interaction between MCs, secondary active transporters (SATs), and cellular processes. This holistic understanding has implications for various fields, from drug discovery to disease pathology.

However, our method requires high-quality, extensive protein sequence data sets, which are not always available, thereby limiting its generalizability. Using a multiple window scanning approach combined with language models, our model has been rigorously tested for generalization. By using diverse window sizes, our model captures local and global sequence patterns effectively, enhancing its adaptability. Advanced language models enhance the model’s contextual understanding, allowing it to generalize outside of training. Across cross-validation as well as independent tests, the model demonstrates consistent performance metrics, demonstrating its ability to accurately classify unknown data. Furthermore, convolutional neural networks and protein language models require substantial computational power, which is prohibitive for insufficient numbers of proteins. The complexity of deep learning models makes it difficult to interpret small groups, which obscures the underlying biological insights. In addition, although our model predicts mitochondrial carriers accurately, it must be validated experimentally before it can be considered reliable. Future research must address these challenges to enhance our computational approach’s robustness and applicability.

## 6 Conclusion

In this study, we present a novel computational approach, MCNN_MC, for the prediction and analysis of mitochondrial carriers (MCs) within a broader class of secondary active transporters. Identification of MCs and elucidation of BA toxicity mechanisms can help us better understand mitochondria in energy metabolism and disease. Combining pretrained protein language models with multiwindow convolutional neural networks, our model demonstrated better performance in identifying MCs, with a particular focus on the ADP/ATP carrier and its interaction with bongkrekic acid.

Our study’s key findings can be summarized as follows:

- The integration of pretrained protein language model embeddings, particularly ProtTrans, significantly enhanced the model’s ability to capture meaningful features and patterns within protein sequences, leading to improved classification accuracy.
- Through systematic evaluation of different window and filter size combinations, we identified the optimal configuration for robust and reliable MC classification performance.
- Our model achieved impressive classification metrics, including a sensitivity of 96.66%, specificity of 95.76%, accuracy of 96.12%, Matthews Correlation Coefficient (MCC) of 91.83%, and an area under the curve (AUC) of 98.55%, outperforming traditional machine learning models.
- The analysis of the ADP/ATP transporters and their interaction with bongkrekic acid has led to novel insights into the molecular mechanisms underlying toxicity, paving the way for therapeutic interventions and mitigation measures.

MCNN_MC represents a significant advance in bioinformatics and protein analysis by providing a powerful tool for identifying and characterizing novel MCs, elucidating their functional roles, and comprehending their involvement in numerous biological processes and disease mechanisms. Using information encoded in protein sequences, our model contributes to the development of targeted drug discovery strategies and precision medicine approaches, particularly in the context of mitochondrial diseases and toxicity-mediated cellular diseases.

## Data Availability

The code and data set are available on Github: https://github.com/B1607/MCNN_MC.git
